# The Other Obesity Epidemic—Of Drugs and Bugs

**DOI:** 10.3389/fendo.2020.00488

**Published:** 2020-07-31

**Authors:** Adonis Sfera, Carolina Osorio, Eddie Lee Diaz, Gerald Maguire, Michael Cummings

**Affiliations:** ^1^Psychiatry, Loma Linda University, Loma Linda, CA, United States; ^2^Department of Psychiatry, Patton State Hospital, San Bernardino, CA, United States; ^3^Department of Psychiatry, Loma Linda University, Loma Linda, CA, United States; ^4^Department of Psychiatry, University of California, Riverside, Riverside, CA, United States

**Keywords:** obesity, psychotropic drugs, Bacteroidetes phylum, xenobiotic sensors, antimicrobials

## Abstract

Chronic psychiatric patients with schizophrenia and related disorders are frequently treatment-resistant and may require higher doses of psychotropic drugs to remain stable. Prolonged exposure to these agents increases the risk of weight gain and cardiometabolic disorders, leading to poorer outcomes and higher medical cost. It is well-established that obesity has reached epidemic proportions throughout the world, however it is less known that its rates are two to three times higher in mentally ill patients compared to the general population. Psychotropic drugs have emerged as a major cause of weight gain, pointing to an urgent need for novel interventions to attenuate this unintended consequence. Recently, the gut microbial community has been linked to psychotropic drugs-induced obesity as these agents were found to possess antimicrobial properties and trigger intestinal dysbiosis, depleting Bacteroidetes phylum. Since germ-free animals exposed to psychotropics have not demonstrated weight gain, altered commensal flora composition is believed to be necessary and sufficient to induce dysmetabolism. Conversely, not only do psychotropics disrupt the composition of gut microbiota but the later alter the metabolism of the former. Here we review the role of gut bacterial community in psychotropic drugs metabolism and dysbiosis. We discuss potential biomarkers reflecting the status of Bacteroidetes phylum and take a closer look at nutritional interventions, fecal microbiota transplantation, and transcranial magnetic stimulation, strategies that may lower obesity rates in chronic psychiatric patients.

**Graphical Abstract:**
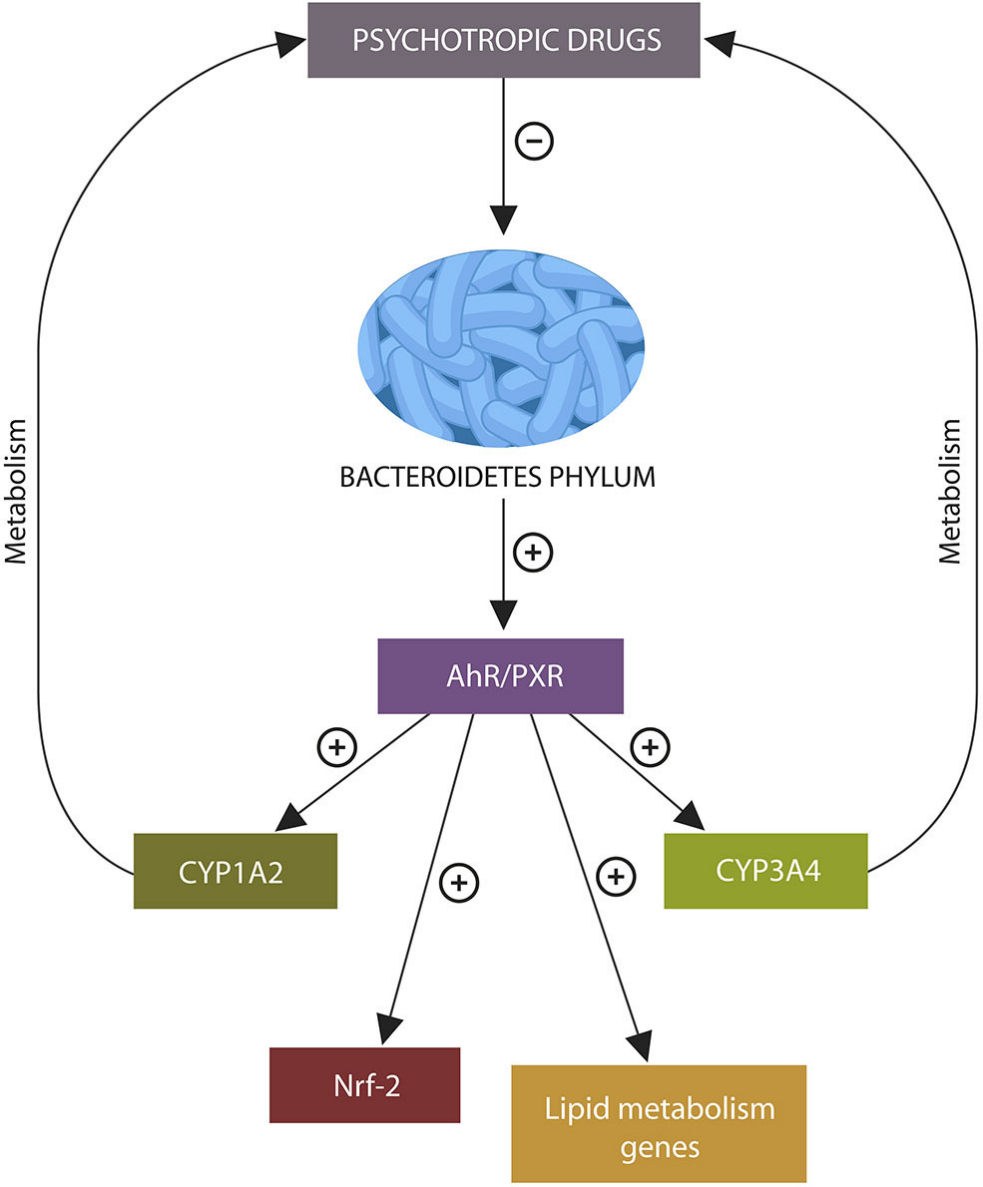
Psychotropic drugs deplete intestinal Bacteroidetes phylum. The absence of metabolites generated by these microbes may under-activate xenobiotic sensors aryl hydrocarbon receptor (AhR) and pregnane X receptor (PXR), disrupting the transcription of genes related to psychotropic drug metabolism, lipid, and redox homeostasis, leading to altered bioavailability of antipsychotic agents, weight gain, and lowered body iron stores.

## Introduction

Chronic psychiatric patients diagnosed with schizophrenia and schizophrenia-spectrum disorders (SSDs), are often treatment-resistant, requiring stabilization with above average doses of psychotropic drugs, especially olanzapine (OLZ), clozapine (CZP), risperidone (RSP), and quetiapine (QTP). Long-term exposure to these atypical antipsychotics (AAPs) increases the risk of metabolic adverse effects and weight gain (1, 2).

Novel studies have identified a bidirectional relationship between the gut bacterial community and psychotropic drugs in which these agents alter the composition of commensal flora while the later influences the pharmacokinetics of the former (3–5). For example, psychotropic drugs-induced depletion of Bacteroidetes phylum may under-activate xenobiotic sensors aryl hydrocarbon receptor (AhR) and pregnane X receptor (PXR), altering the expression of P450 isozymes CYP1A2 and CYP3A4 that are involved in the metabolism of these agents (6). Aside from the CYP system, gut microbes express numerous phase I and II enzymes that were shown to transform various drugs prior to absorption (7). On the other hand, agents conjugated in the liver and secreted in the bile can be deconjugated by gut microbes, increasing their toxicity (8). These novel findings led to the development of pharmacomicrobiomics, a new field investigating the complex interactions between the drugs and gut microbes (9).

Historically, psychotropic drugs have been known to possess antimicrobial properties but their role in causing dysbiosis was only appreciated after the microbiome discovery. For example, most studies in humans show that AAP-treated patients develop an obesogenic microbial pattern, marked by increased Firmicutes and Actinobacteria phyla and decreased Bacteroidetes (10, 11). Interestingly, AAP-upregulated phyla have the capability to synthesize heme, likely enabling them not only to survive but also thrive in iron-scarce conditions due to nutrients left behind by the non-surviving competitors.

Preclinical research offers more insight into the psychotropics-obesity link via germ-free animals that proved crucial for the study of AAP-related dysbiosis. Given that these studies are based on rodents, it is unclear at this time if the findings can be translated to humans, however body weight alteration have been minimal in germ-free rodents exposed to AAPs (12). Others have reported that prebiotic treatment can attenuate OLZ-induced weight gain in mice, further underlining the role of commensal flora in lipid homeostasis (13, 14). Another study has reported that antibiotic pretreatment prevented weight gain in OLZ-treated female rats, emphasizing once more the role of enteric microbiota in obesity (15). Several recent studies have found that, aside from antipsychotics and antidepressants, several other drugs possess antimicrobial functions and induce dysbiosis (16–19).

Taken together, there is a rapidly growing body of evidence showing that some psychiatric treatments can alter the composition of gut microbiota, triggering dysbiosis and dysmetabolism. On the other hand, intestinal microbes can alter the absorption and pharmacokinetics of psychotropic agents, impairing their bioavailability. Conversely, restoring microbial number and diversity by diet, pre or probiotics may attenuate or prevent psychotropic drugs-associated weight gain.

In this paper, we discuss the role of microbiota in the metabolism of psychotropic drugs as well as the dysbiosis associated with the antimicrobial properties of these agents. We suggest potential biomarkers, of Bacteroidetes phylum and take a closer look at various interventions, including dietary fiber, fecal microbiota transplant (FMT) and transcranial magnetic stimulation (tMS) in preventing weight gain associated with psychotropic agents.

## Psychotropic Drugs and Obesity

There is little doubt that obesity has increased worldwide and reached epidemic proportions, engendering one of the most complex public health problems faced by the society today (20, 21). However, it has been less emphasized that another obesity epidemic of even greater proportions has been taking place silently in mentally ill patients, placing them at higher morbidity and mortality risk compared to the general population (22). Indeed, obesity rates and cardiovascular disease are 2–3 times higher in psychiatric patients, especially in women, children, and adolescents (23–26). Historically, reports of weight fluctuations and abnormal eating behavior in mentally ill individuals have been observed prior to the psychotropic drugs era, suggesting that either psychiatric disorders cause dysmetabolism or impaired metabolism leads to abnormal brain functioning (27).

There are several excellent reviews on weight gain and obesity in psychiatric patients, discussing receptor interactions, orexigenic peptides, insulin resistance, and reward mechanisms (28, 29). These topics are beyond the scope of this article that focuses primarily on the impact AAP drugs on gut microbial community.

Over the past few years, several studies have emphasized the role of gut microbial community in obesity and dysmetabolism as it was noted that germ-free animals exposed to psychotropics do not display weight fluctuations (12). This led to a heightened interest in microbial metabolites and their role in engendering an immunologically tolerant enteric environment optimal for nutrient harvesting. On the other hand, dysbiosis or selective elimination of microbiota by xenobiotics was linked to local pathology, and systemic disorders, including obesity and psychiatric conditions (30, 31). Interestingly, both psychotropic drugs-induced dysmetabolism and high fat diet-related weight gain present with a common enteric microbial pattern, depletion of Bacteroidetes phylum, suggesting an overlapping pathology. Others have opined that the loss of Bacteroidetes-generated metabolites is the common denominator of weight gain induced either by an unhealthy diet or psychotropics drugs (6, 32). Therefore, restoring the levels of these molecules may lead to novel weight loss strategies in chronic psychiatric patients.

### The Holobiont, a Story of Two Kingdoms

The concept of the holobiont refers to a holistic model of two biological kingdoms living together: the eukaryote host and prokaryote microbes. This coexistence is made possible by the inter-kingdom cross talk via dietary molecules and microbial metabolites, enabling immunological tolerance of the microbial organ and food, while maintaining vigilance for pathogens, toxins, and pollutants (33–35). To accomplish all these tasks, nutrient harvesting is highly intertwined with immunity and xenobiotic metabolism through a system of promiscuous sensors expressed by intestinal epithelial cells (IECs) and enteric macrophages, such as AhR and PXR (36). These xenobiotic receptors activate innate immunity proportionally with the affinity of their binding ligands, rather than as binary “on-off” switches. For example, a strong agonist may trigger immune activation, while partial or weak agonists may induce immunosuppression (37). Indeed, high affinity AhR ligands have been canonically linked to the immune rejection of toxins and pollutants (38). However, the recent discovery of endogenous AhR and PXR ligands with weak or partial agonism synthesized by the gut microbes from dietary tryptophan (Trp) and undigestible fiber has shed some light on the role of xenobiotic sensors in both immune tolerance and energy metabolism (39). Indeed, loss of endogenous AhR ligands was associated with weight gain and decreased IL-10 that impaired commensals immune tolerance (40–43). By the same token, loss of microorganismal variety with depletion of microbial phyla was linked to both immune rejection of commensals and weight gain (44).

### Of Kingdom Food Tasters and Cupbearers

Gut microbiota ensure their own acceptance into the GI habitat by generating weak AhR and PXR ligands, including indole-3-acetate and indole-3-propionate (IPA) that promote immunological tolerance by upregulating IL-10 (45–47). At the same time, microbiota assist the host by filtering xenobiotics, toxins, pollutants, and heavy metals as well as by denying nutrients to pathogenic bacteria (48). Therefore, gut microbes mediate not only their own immunological tolerance but also that of dietary molecules by converting them into AhR and PXR ligands (49).

The exact molecular underpinnings of immunological tolerance to microbes and dietary molecules are unclear at this time, however a recent study identified toll like receptor 9 (TLR9)-AhR signaling as the immunosuppressive switch that turns off innate immunity in the presence of apoptotic cells, suggesting that a similar mechanism may engender the tolerance of enteric microbes and food (50). Indeed, TLR9 is an established tolerogen that upregulates IL-10 during pregnancy, promoting fetal acceptance (51).

Taken together, these studies suggest an immunosuppressive TLR9-AhR-IL-10 axis of tolerance likely involved in the acceptance of gut microbes and food. Furthermore, TLR-9 was demonstrated to sense eukaryotic and prokaryotic DNA and contribute to lipid homeostasis, connecting genomic damage to obesity (52, 53). Indeed, it has been established that iron and related reactive oxygen species (ROS) induce DNA disruption with resultant dysmetabolism (54).

### The Kingdom Messengers

Microbiota-derived molecules, including propionate, indole, neurotransmitters, and iron-related molecules, including bacterial siderophores and ROS were demonstrated to signal with the receptors expressed on the intestinal epithelial cells (IECs) and enteric macrophages, modulating both feeding and immunity. The following is a snapshot of inter-kingdom signals relevant for psychotropic drugs-induced weight gain.

### Propionate

**Figure 1:**
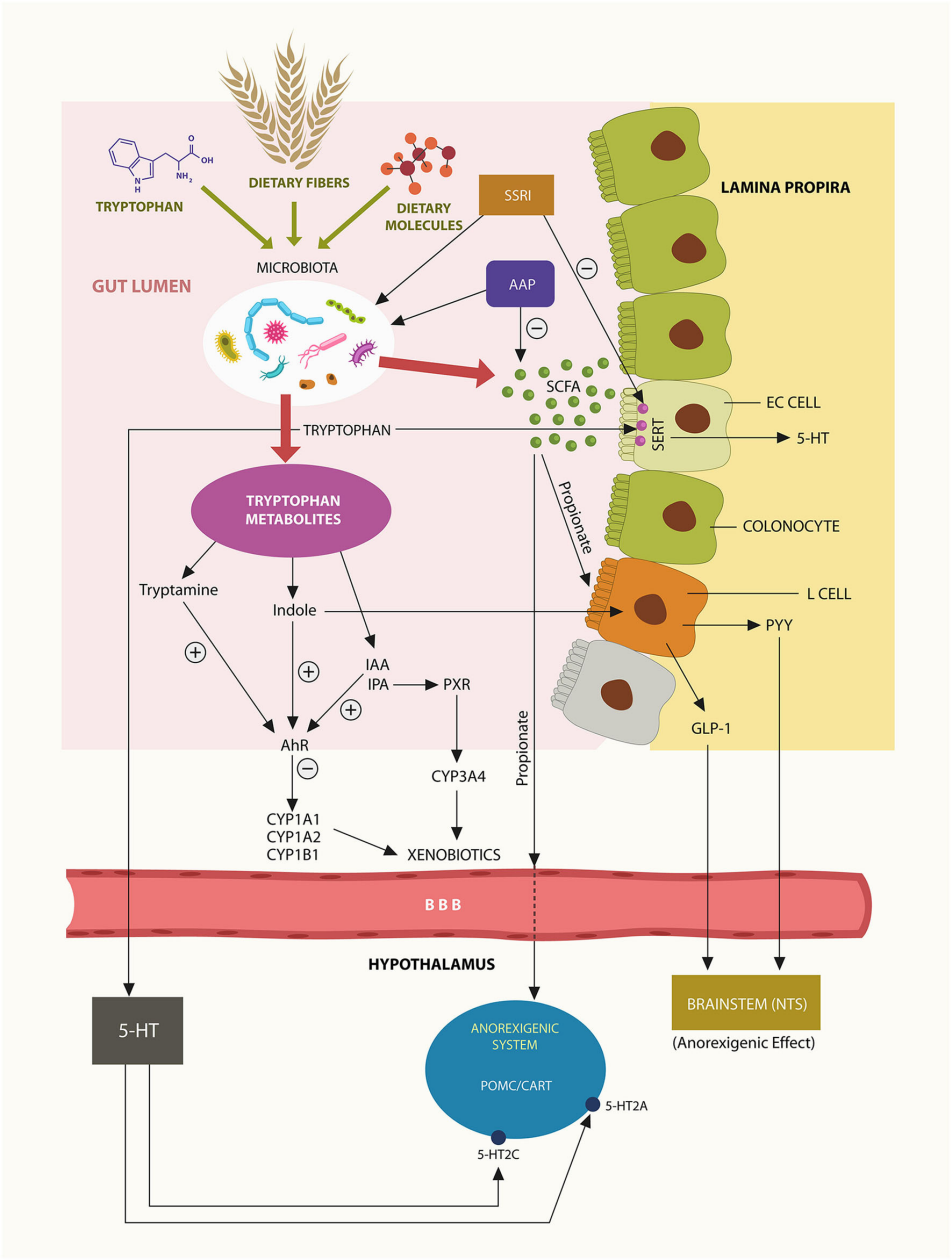
Trp, dietary fiber and food are converted by gut microbes into ligands at AhR and PXR. Bacteroidetes phylum generates most enteric propionate, inducing L-cells activation and release of anorexigenic peptides GLP-1 and PYY that lower appetite via NTS. Propionate may also cross the BBB and act on hypothalamic POMC/CART neurons, inhibiting feeding behavior. Trp metabolite, indole is an AhR agonist, while IPA is a PXR agonist. Activation of these xenobiotic sensors induces the transcription of psychotropic drugs metabolizing enzymes, including CYP1A2 and CYP3A4. A small fraction of gut Trp crosses into the brain, generating the central 5-HT.

SCFAs, acetate, propionate, and butyrate, are generated by gut microbes via fermentation of dietary fiber. These molecules activate host intestinal G protein-coupled receptors (GPCRs), including GPR41 and GPR43, modulating numerous physiological functions, including feeding and immunity (55, 56). Bacteroides phylum is the major generator of enteric propionate, a SCFA that interacts with the Trp metabolite indole, engendering indole-3-propionic acid (IPA) a biomolecule involved in lipid homeostasis and acceptance of commensal microbes (57). In addition, propionate is a potent agonist at GPR41 and GPR43 expressed by enteric L-cells, enabling the release of anorexigenic hormones, glucagon-like peptide-1 (GLP-1), and peptide YY (PYY) (Figure 1). These bioactive molecules were demonstrated to lower appetite and feeding by activating the nutrient sensing neurons in brainstem nucleus tractus solitarius (NTS) (58) (Figure 1). Indeed, clinical trials with human subjects have demonstrated that propionate lowers food intake and increases energy expenditures via NTS (59, 60). As Bacteroidetes phylum generates most of the GI tract propionate, psychotropic drugs-associated depletion of these microbes may lower propionate, triggering dysmetabolism and weight gain. For example, a recent study in non-psychiatric, obese individuals associated propionate with weight loss (59). This study suggests that Bacteroidetes depletion, including that induced by psychotropic drugs, may trigger dysmetabolism and weight gain (59). It is, therefore realistic to expect that propionate fecal levels may accurately reflect the status Bacteroidetes phylum (**Figure 3**).

Aside from its local physiological functions, propionate may cross the blood-brain barrier (BBB) to act on pro-opiomelanocortin (POMC) and cocaine-and amphetamine-regulated transcript (CART) hypothalamic neurons, suppressing food intake (61). Moreover, the serotonin (5-HT) precursor Trp was demonstrated to cross the BBB and, upon conversion to 5-HT, activate POMC/CART, 5-HT2A, and 5-HT2C receptors, modulating appetite (62) (Figure 1).

### Tryptophan Metabolites

Trp is an essential amino acid that cannot be synthesized in the human body therefore it must be obtained entirely from the diet. In the gut, Trp is converted by microbes, including Bacteroidetes into AhR and PXR ligands that promote weight loss and immune tolerance to commensals (57, 63).

Under normal circumstances, Trp is converted in the GI tract to indole by tryptophanase-producing bacteria, including Bacteroidetes thetaiotaomicron (B. theta) a member of Bacteroidetes phylum. Since indole is a metabolite associated with weight loss, depletion of Bacteroidetes may lead to obesity (64, 65). Moreover, others have found that, like propionate, indole can also activate enteric L-cells, releasing the anorexigenic peptide hormones GLP-1 and PYY, further lowering body weight (66) (Figure 1).

The GI tract 5-HT does not cross the BBB but plays a major local role as it promotes intestinal motility (67). Interestingly, IECs express serotonin transporters (SERT), proteins inhibited by serotonin reuptake inhibitors (SSRI), explaining the beneficial role of these agents in Crohn's disease and ulcerative colitis (68–70). As shown above, Trp and propionate participate in the production of IPA, a PXR ligand associated with immune tolerance and weight loss (71–73).

### Neurotransmitters

Aside from 5-HT, intestinal commensal flora is known for synthesizing many neurotransmitters, including dopamine (DA), norepinephrine (NE), and acetylcholine (ACh) that can act locally, but are also believed to reach the CNS and influence brain physiology (74–76). These biomolecules play a key role in energy metabolism and immune tolerance of gut microbes by upregulating IL-10 (77, 78). Interestingly, microbial DA and NE were demonstrated to influence iron absorption and metabolism by functioning as siderophores or bacterial iron scavengers (79, 80). Interestingly, a recent study found that enterobactin, an *Escherichia coli* siderophore, can be transferred to the host, revealing a novel form of inter-kingdom communication (81). On the other hand, Bacteroides phylum does not generate siderophores, but seems to “borrow” them from other microbes, including *E. coli* (82). Aside from enterobactin, *E. coli* was demonstrated to provide Bacteroidetes with porphyrins, emphasizing this phylum's dependence on iron and heme (83). This also demonstrates a special symbiosis and interdependence between Bacteroidetes and *E. coli* (83). Since psychotropic drugs were demonstrated to eliminate both microbial groups, the porphyrin exchange may be impaired, resulting in lower iron stores (83). Indeed, recent studies have indicated that enterobactin possesses antioxidant properties and may contribute to host redox homeostasis (84). Moreover, IPA was demonstrated to exert antioxidant properties against iron-generated ROS, reversing metabolic disturbances associated with genomic damage (54, 85). Taken together, it would be interesting to study if psychotropic drugs-induced oxidative stress may lead to weight gain associated with these agents.

### Iron and ROS

The connection between psychotropic drugs and iron has been previously documented as iron deficiency anemia, restless leg syndrome, tardive dyskinesia, akathisia, and neuroleptic malignant syndrome were associated with iron dysmetabolism (86–89). Moreover, low iron levels were linked to the negative symptoms of schizophrenia, while the offspring of hypoferremic mothers were found to be at risk of developing this disorder (90). Nutritional immunity, the shifting of iron from the extracellular to the intracellular compartment to withhold it from pathogens during infection, may be the mechanism, linking the motor adverse effects of psychotropic drugs to low extracellular iron (86, 88, 91). Indeed, since nutritional immunity leads to excess intracellular iron, placing this biometal in close proximity to lipids, the risk of lipid peroxidation and ROS generation is increased (92). Moreover, psychotropic drugs modulate lipid metabolism, regulating iron absorption and ROS generation (93). For example, Iron depletion was demonstrated in RSP-treated children and adolescents, possibly indicating iron shifting from the extracellular to the intracellular compartment via nutritional immunity (94).

In humans, heme and non-heme iron are absorbed at different GI tract locations as the uptake of the later occurs in the proximal small bowel where it is regulated by hepcidin, while the former is very efficiently absorbed in the colon (95). Bacteroidetes-generated propionate facilitates the absorption of iron, linking depletion of this phylum with iron dysmetabolism (15, 96–98). Indeed, as mentioned above, decreased host iron stores were previously reported in connection with psychotropic drugs (86).

In summary, several psychiatric medications may act as indirect iron chelators as they lower the absorption of this micronutrient via Bacteroidetes phylum depletion.

## Gut Microbes and Psychotropic Drugs Metabolism

Cytochrome P450 enzymes play a key role in the metabolism of xenobiotics. These iron-containing cytochromes are expressed in both intestinal tissue and the liver, however the enteric systems require more research as their specifics are not clear at this time. Compounding this problem is the fact that gut microbes express as many as 3000 cytochrome P450 (CYP450) enzymes that transform most drugs and dietary molecules the host consumes (99).

The major host intestinal cytochrome, CYP3A4 metabolizes over 50% of common pharmaceuticals, while about 20% of clinically used drugs are metabolized by CYP1A2 (100, 101). For example, CLZ and OLZ, are processed by CYP1A2, RSP is partially metabolized in CYP3A4, while QTP is converted to N-desalkylquetiapine in CYP3A4 (102–105). As xenobiotic sensors AhR and PXR control the expression of CYP1A2 and CYP3A4, respectively, the activation status of these receptors can alter the bioavailability of the drugs metabolized by these cytochromes.

AhR is a ligand-activated receptor, complexed in the IECs cytosol with HSP90 protein. Upon AhR ligand-binding, the entire complex migrates to the nucleus where it activates AhR nuclear translocator (ARNT), enabling the transcription of many genes, including CYP1A1, CYP1A2, and CYP1B1, several lipid homeostasis genes and the Nrf-2 gene (37, 106–109). Nrf-2, itself a transcription factor, controls the expression of numerous other genes, including those encoding for iron storage proteins and phase I and II drug-metabolizing enzymes, associating this redox protein with drug metabolism and iron dyshomeostasis (110, 111).

**Figure 2:**
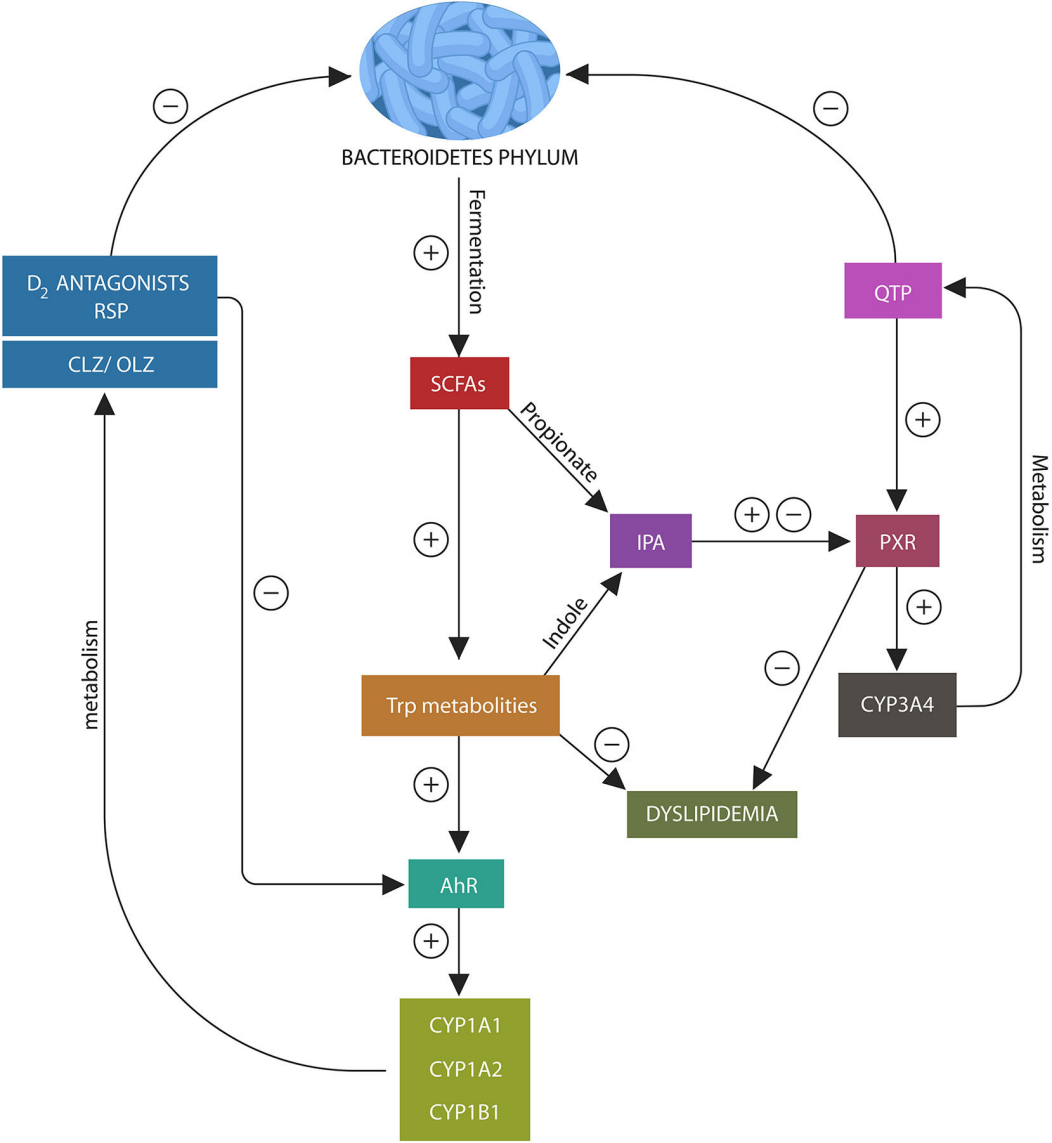
Psychotropic drugs deplete Bacteroidetes phylum as do most D2 blockers. This lowers the AhR and PXR ligands, propionate and indole, under-activating these xenobiotic sensors. This in turn, inhibits the expression of CYP1A1/A2, CYP1B1, and CYP3A4, impairing psychotropic drugs metabolism. In addition, loss of AhR and PXR ligands disrupts the expression of several lipid homeostasis genes and Nrf2 (not shown), leading to weight gain and impaired iron metabolism.

Bacteroidetes phylum activates AhR by generating propionate which forms AhR ligands with indole. Depletion of these microbes by psychotropic drugs may alter the bioavailability and metabolism of these agents (10). For example, OLZ-induced Bacteroidetes phylum depletion and lower propionate, may under-activate AhR, suppressing the expression of CYP1A2 gene. Since OLZ metabolism is dependent on the CYP1A2 gene, its inhibition can alter the metabolism and bioavailability of this drug (Figure 2) (105).

**Figure 3:**
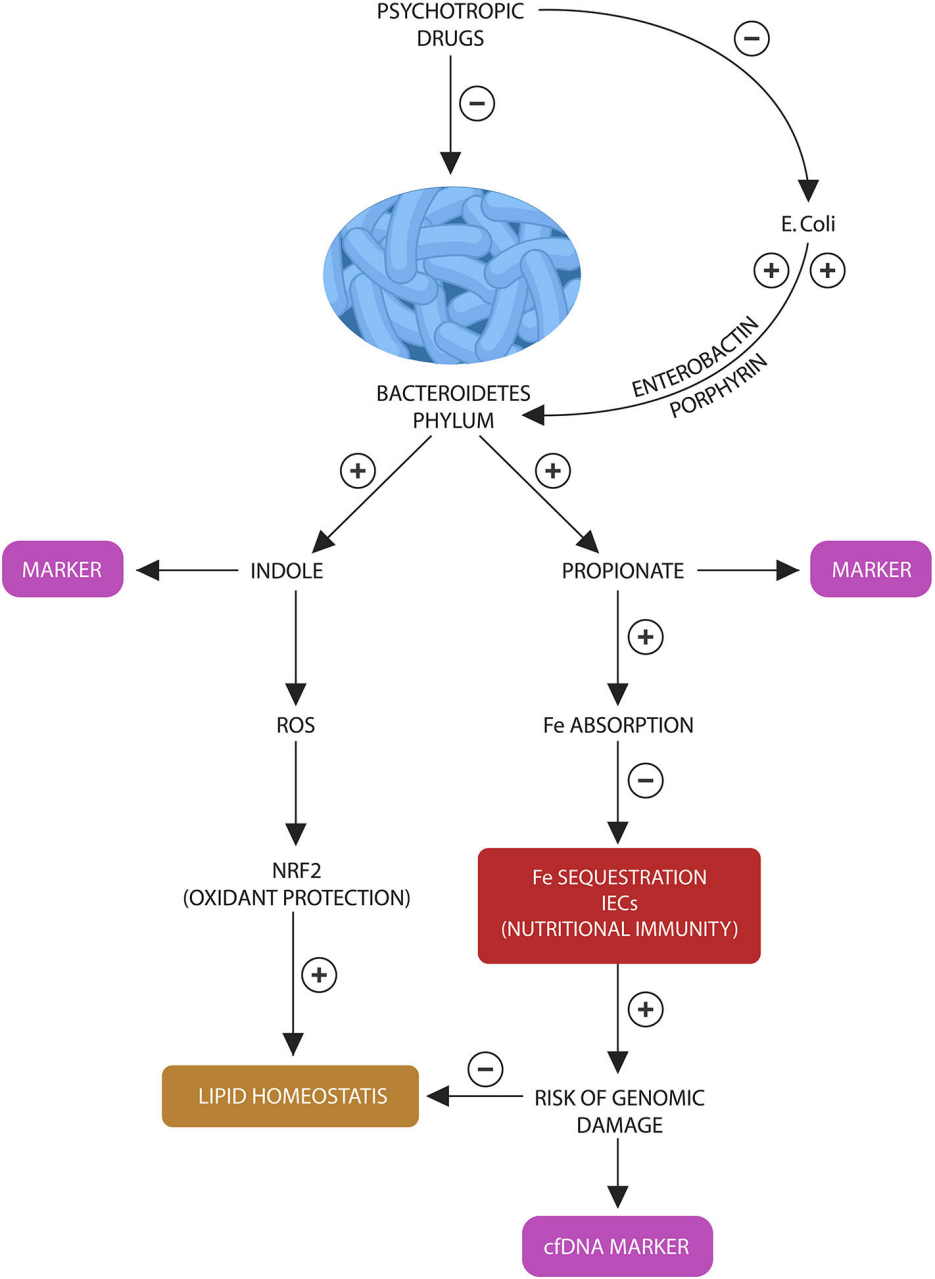
Bacteroidetes stress test: Bacteroidetes phylum and *E. coli* participate in a “special” symbiosis in which the later provides the former with iron-molecules enerobactin and porphyrins. Bacteroidetes in return generate indole and propionate (suggested as fecal biomarkers). In various combinations, indole and propionate are AhR and PXR ligands, contributing to lipid and redox homeostasis. Propionate also facilitates iron absorption, preventing the activation of nutritional immunity in intestinal macrophages and IECs. Lowered propionate (due to Bacteroidetes depletion) triggers nutritional immunity with iron sequestration, increasing the risk of ROS and genomic damage, measured by the cfDNA marker.

In the same manner, PXR receptor ligands enable the migration of this xenobiotic sensor to the nucleus from where it facilitates the expression of CYP3A4 and several lipid homeostasis genes (112–114) (Figure 3). Since QTP, metabolized by CYP3A4, is a potent PXR agonist, it may alter both its own and lipid metabolism, probably explaining the weight gain associated with this drug (115). Moreover, QTP, like other psychotropics, can induce gut dysbiosis and lower IPA biosynthesis, thus suppressing CYP3A4 gene and altering its own metabolism (Figure 2). A similar mechanism may be at work during the biotransformation of CLZ which is partially metabolized by CYP1A2. Bacteroidetes depletion and AhR under-activation may impair CLZ bioavailability and blood levels (105, 116). Moreover, recent studies in rodents found that psychotropic drugs can inhibit AhR directly by antagonizing D2 receptors, impairing the expression of CYP1A1, CYP1A2, and CYP1B1 (117) (Figure 2). Indeed, as most antipsychotic drugs are D2 blockers, they may lower their own metabolism both directly and via dysbiosis. Interestingly, valproic acid (VPA), a natural, branched short-chain fatty acid, was associated with intestinal dysbiosis as it also depletes Bacteroidetes, probably explaining the weight gain associated with this drug (118, 119). In addition, VPA was shown to augment the expression of intestinal and hepatic CYP3A4, indicating that this drug may alter its own metabolism via dysbiosis (120). These novel findings are relevant as they underline not only the importance of gut flora in drug metabolism but also the need for obtaining psychotropic blood levels in clinical practice.

Taken together, the new science of pharmacomicrobiomics explains the role of the microbial organ in psychotropic drugs metabolism, underlining the interconnectedness between xenobiotic metabolism and nutrient harvesting.

## Psychotropic Drugs As Antimicrobials

The awareness that psychotropic drugs have antimicrobial properties goes back to the nineteenth century when Paul Ehrlich observed the bactericidal action of methylene blue, a molecule that led the synthesis of chlorpromazine, ushering the development of antipsychotic agents (121). Moreover, antidepressant drugs can be traced back to the antimicrobial isoniazid which was found to induce euphoria in tuberculosis patients (122). For this reason, it should not be surprising that many psychotropic agents retain antimicrobial properties to this day. However, with the advent of the microbiome, these historical data have recaptured the attention of researchers and clinicians, prompting some to link chronic psychosis to infection, immunity, and antimicrobial treatments (123).

Most studies in humans have found that Bacteroidetes phylum (that together with Firmicutes comprise 90% of gut microbiota) is more vulnerable to psychotropic drugs than other phyla (6, 124). For example, chronic RSP treatment in children was associated with lower fecal Bacteroidetes compared to antipsychotic-naïve controls (97). This is surprising since Bacteroidetes are well-known for antibiotic-resistance by virtue of expressing numerous drug efflux pumps (DEPs) that facilitate the expulsion of drugs from the intracellular compartment (125). Interestingly, several psychotropic drugs, including the antipsychotic loxapine, phenothiazines, and selective serotonin reuptake inhibitors (SSRIs) were found to effectively block microbial DEPs, probably explaining their selective bactericidal action against Bacteroidetes phylum (126–128). Moreover, iron chelators were recently found to be effective DEP-inhibitors, a property that may explain their bactericidal action against the iron-dependent Bacteroides phylum (129). Indeed, another reason, Bacteroidetes phylum may be more susceptible to psychotropic drugs is that, unlike Firmicutes and Actinobacteria that can synthesize heme, Bacteroidetes, which lack the enzymatic machinery to synthesize this iron protein, depend on scavenging it from the colonic environment (82, 130, 131). Conversely, as Bacteroidetes phylum generates propionate that facilitates iron absorption, their depletion may impact host iron stores (12, 15, 97, 132). In addition, impaired iron absorption may activate nutritional immunity in IECs and macrophages to sequestrate this biometal and deny it to pathogens, exacerbating hypoferremia and metabolic syndrome (133, 134). On this point, a novel study in children and adolescents chronically treated with RSP found that iron status was inversely corelated with weight gain, contributing to dysmetabolic iron overload syndrome (DIOS) (135). This is significant as intracellular iron increases the risk of ROS generation that cannot be counteracted as Bacteroidetes depletion lowered IPA, a key gut antioxidant (98, 136, 137). Restoration of the adequate levels of AhR and PXR ligands via diet, pre, probiotics, or microbial transplant may upregulate Bacteroidetes phylum, attenuating psychotropic drugs-induced weight gain (138).

Taken together, recent studies seem to suggest that the anti-DEP properties of psychotropic drugs may selectively deplete Bacteroidetes phylum as these microorganisms express abundant DEPs and rely on these proteins for antibiotic defense. Conversely, depletion of Bacteroidetes phylum may impair iron and heme absorption, leading to DIOS.

### Psychotropics, the New Antibiotics?

Animal studies have contributed further to the understanding of dysbiosis induced by the antimicrobial properties of psychotropic drugs. For example, a recent study reported that RSP-treated female mice exhibited significant weight gain and altered gut microbial diversity; however, when these rodents received a fecal transplant from RSP-naïve mice, the weight gain was reversed, underlining the role of commensals in obesity (97). Another preclinical study demonstrated that OLZ completely inhibited the growth of *E. coli in vitro*, emphasizing that the spectrum of this agent's antimicrobial properties extends beyond Bacteroidetes (12). On the other hand, antibiotics co-administered with OLZ, improved metabolic parameters, resulting in weight loss (15). It appears that a significantly reduced gut bacterial content, resembling germ-free status, is necessary to prevent psychotropics-induced weight gain, while a less drastic reduction in microbial number, has the opposite effect (19). A different study showed reduced gut commensal flora diversity in mice chronically treated with antidepressants. Interestingly, this study also found that excessive *Ruminococcus flavefaciens* can attenuate the efficacy of duloxetine, a serotonin-norepinephrine reuptake inhibitor (SNRI), suggesting microbiota involvement in the metabolism of this agent (139).

Other studies have reported that AADs are not the only psychotropics with antimicrobial activity as fluoxetine, sertraline, and escitalopram were reported to induce gut dysbiosis (6). For example, fluoxetine has demonstrated antimicrobial activity against Gram-positive microbes, including Staphylococcus, Enterococcus, and anaerobes such as *Clostridium difficile* and perfringens (140). Interestingly, as opposed to AAP drugs that affect primarily Gram negative Bacteroidetes, SSRIs appear to deplete Gram positive bacteria. Moreover, a novel study associated fluoxetine treatment with multi-antibiotic resistance via iron and ROS-induced DNA damage (141). Indeed, epidemiologic studies have associated long-term antidepressant treatment with ROS-induced obesity, connecting once more iron dysmetabolism with weight gain (142, 143).

The antimicrobial action of antipsychotic drugs against DEP-expressing bacterial strains has rendered these agents excellent candidates for antibiotic resistant bacteria (144). Indeed, a recent study found that phenothiazines, as a group are DEP-inhibitors, effective against several drug resistant bacterial strains, while another study found trifluoperazine beneficial to the treatment of sepsis (145, 146). In addition, QTP and OLZ were found effective against fungal DEPs and are currently being tested as anti-Cryptococcal drugs (147). Conversely, long-term treatment with antidepressant or antipsychotic drugs was associated with increased risk of methicillin-resistant Staphylococcus aureus (MRSA), further linking these agents to both the therapeutic and adverse effects of antibiotics (148). This is significant because chronic psychiatric patients with schizophrenia and SSD are frequently treatment-resistant and often require long-term use of high doses of psychotropic drugs to remain asymptomatic. Prolonged exposure to these agents increases the risk not only of weight gain and cardiometabolic disorders but also of antibiotic resistance, resulting in poorer outcomes and higher medical cost. Moreover, aside from psychotropics, several drug categories, including proton pump inhibitors (PPIs), histamine-2 (H2) blockers, antimitotic agents and non-steroidal anti-inflammatory drugs (NSAIDs) were reported to induce both dysbiosis and iron dysmetabolism, indicating the need for medical and psychiatric care integration (17, 86, 149, 150).

## Markers and Interventions

Although life-changing for patients, psychotropic medications have been accompanied by an unintended consequence, an obesity epidemic that has increased the mortality, morbidity, and medical expenditures in this population (151). Fortunately, weight gain is a modifiable risk factor and a better understanding of its pathogenesis vis-à-vis psychotropic drugs may lead to the development of novel preventive strategies. Moreover, since preclinical studies have connected gut dysbiosis with the weight gain associated with psychotropic drugs, biological markers reflecting the health of gut microbes are of primary importance. For example, fecal propionate levels may mirror the status of Bacteroidetes phylum in general as these microbes are the major propionate producers. On the other hand, indole levels may be more species specific, reflecting Bacteroides thetaiotaomicron, that specializes in indole production. Since psychotropic drugs deplete the entire Bacteroidetes phylum, propionate, and indole levels may provide an index of microbial integrity. In addition, we propose a non-invasive peripheral blood marker, consisting of cell free DNA (cfDNA) with the specific Bacteroidetes motif, GTCGTT as an assessment tool for the psychotropic drugs-induced Bacteroidetes depletion. Aside from the markers of microbial loss, various nutrients and procedures, including fiber, probiotics, fecal microbial transplant, and vagus nerve manipulation were found helpful for preventing weight gain and restore physiological levels of Bacteroidetes phylum.

### Beware of Stressed Bacteroidetes

Extracellular or cfDNA, a marker of eukaryotic and prokaryotic genomic damage, was demonstrated to strongly activate TLR9, an action that promptly turns off immunological tolerance to gut microbes and activates vigilance to potential pathogens (152). Recent studies have shown that cfDNA is released into the blood following eukaryotic or prokaryotic cell death, comprising a marker of microbial or host cells demise (153). Moreover, others have shown that bacterial DNA derived from Gram negative microbes, including Bacteroidetes, activates TLR9 more robustly than Gram positive DNA, suggesting that this receptor links immune activation to the body weight via AhR signaling (154). Furthermore, it has been established that Bacteroidetes DNA is marked by an overabundance of the genomic motifs GTCGTT, strong TLR9 activators, indicating that cfDNA carrying this sequence is a not only a hallmark of this phylum but also an influencer of body weight via TLR9-AhR-IL-10 signaling (155). For this reason, we construe that elevated GTCGTT cfDNA in peripheral blood may accurately reflect the risk of both psychotropic drugs-associated Bacteroidetes depletion and weight gain (156). Therefore, we propose a combined non-invasive peripheral blood test: real-time polymerase chain reaction (PCR) to detect cfDNA and CpG oligodeoxynucleotide (ODN) to identify GTCGTT motifs (157, 158).

We also believe that cfDNA alone, without the Bacteroidetes marker, may accurately mirror the activation of host nutritional immunity, iron sequestration and ROS-induced genomic damage in IECs and enteric macrophages. This probably takes place as a result of Bacteroidetes depletion as this phylum facilitates iron absorption by generating propionate (Figure 3). Insufficient iron may activate host nutritional immunity to preserve this biometal intracellularly, however chronic iron retention increases the risk of ROS and DNA damage, resulting in l cfDNA release. For this reason, we believe that cfDNA may accurately measure nutritional immunity, iron sequestration and genomic damage. Indeed, cfDNA is currently a marker of iron-induced DNA disruption in hemodialysis patients (159–161). Furthermore, since inter-kingdom signaling via ROS-Nrf2 mediate antioxidant and lipid homeostasis, deficient body iron stores may reflect the risk of weight gain (162, 163). Indeed, DIOS is encountered in many overweight individuals, indicating that excessive ROS generation may promote obesity (132). In addition, RSP-treated patients have demonstrated not only depleted iron stores but also weight gain proportional to hypoferremia (86).

In conclusion, to assess the integrity of host-microbiota interface, we propose a battery of four tests: peripheral blood cfDNA to reflect the status of host nutritional immunity, GTCGTT cfDNA to assess Bacteroides depletion along with fecal indole and propionate to further estimating the depletion of this phylum (Figure 3). We believe that the following laboratory assays can be optimal for detecting indole and propionate: hydroxylamine-based indole assay (HIA) as it was recently reported to accurately detect indole in complex clinical samples, including feces and liquid chromatography tandem mass spectrometry (LC-MS/MS) based on 3-nitrophenylhydrazone (3NPH) derivatization, an assay shown to accurately measure fecal propionate (164, 165).

### Dietary Fiber as a Prebiotic

The hallmarks of Western diet, low fiber content, high refined sugars and fats, suggest that these nutrients, along with physical inactivity may, at least in part, account for the modern obesity epidemic (166). Novel studies have found that the general population of Western countries consumes an average of 10–20 g of dietary fiber per day (35–50 g being optimal), while much lower fiber intake characterizes the diet of chronic psychiatric patients (167–170). Both dietary fiber and fermenting microbes are necessary for the generation of indole and propionate. However, fiber was found to possess probiotic properties by promoting the growth of fermenting microbes, including Bacteroidetes (60, 171, 172). Aside from fiber, direct supplementation with sodium propionate was shown to increase energy expenditure and protect against high fat diet-induced obesity in rodents, suggesting that fiber or its derivative, propionate may reverse dyslipidemia in chronic psychiatric patients. However, prior to clinical use, more studies on sodium propionate are needed to assess its safety in humans. Fiber supplementation or high fiber diets, on the other hand, should be used routinely in chronic psychiatric patients (173).

### Probiotics to Restore the Levels of Propionate

Probiotics or microbes beneficial to health have recently reawaken the interest of researchers and clinicians as several bacterial species, including *Bacteroides fragilis* and *Bacteroides uniformis* may selectively upregulate gut propionate levels (174, 175). For example, in preclinical studies, supplementation with *B. uniformis* was found to restore immunological and metabolic homeostasis caused by intestinal dysbiosis, indicating potential benefit for chronic psychiatric patients (176). Furthermore, indole-producing Bacteroides thetaiotaomicron has shown anti-obesity benefits in preclinical studies but to our knowledge, it has not been tested in humans (177). For this reason, more studies are needed as this microbe presents with the unique property of converting succinate to propionate (175). Since circulating succinate is increased in hypertension, ischemic heart disease, and type 2 diabetes, conditions prevalent in chronic psychiatric patients, a probiotic converting this molecule into the anorexigenic propionate is very promising (178).

### Fecal Microbial Transplant (FMT) for Indole and Propionate Augmentation

FMT is an ancient procedure that has been practiced throughout the centuries to alter the balance of colonic microbes and protect against infections. FMT is commonly used in veterinary medicine, while in humans, it is currently approved for the treatment of *Clostridium difficile* infections (179, 180). The role of FMT in microbial dysbiosis-linked chronic disorders is still in the early stages and requires more studies in humans, specifically for developing adequate criteria for the identification of optimal donors (181). Based on findings from preclinical and small clinical studies, FMT may provide benefits in IBD and is currently being investigated for the treatment of several chronic conditions, including obesity and metabolic disorders (182, 183). We believe that when obtained from the adequate donors, FMT may have the potential to restore the physiological levels of propionate and indole in chronic psychiatric patients.

### Deep Transcranial Magnetic Stimulation (dTMS) and Intermittent Vagal Blockade (vBloc)

Studies on dTMS, repetitive transcranial magnetic stimulation (rTMS) and transcranial direct current stimulation (tDCS) to decrease food intake and weight are currently ongoing but further data is needed before recommending these procedures to chronic psychiatric patients (180, 184, 185). In 2015, the U.S. Food and Drug Administration approved a vagal blocking device (vBloc) for the treatment of moderate to severe obesity, linking vagus nerve to feeding behavior (186). Interestingly, both intermittent vagal blockade and vagal stimulation were found effective for lowering body weight, suggesting an indirect effect on metabolism, probably mediated by gut microbes (187, 188). Moreover, a peripheral anti-inflammatory vagal system, operating via alpha 7-cholinergic nicotinic receptors on peripheral macrophages, has been known to dampen inflammation, suggesting that nicotinic signaling may also modulate the enteric microbes via a similar system (189, 190). Indeed, microbiota-derived acetylcholine (ACh) has been recently reported, suggesting ongoing cholinergic signaling between the microbial organ and vagus nerve (191). Interestingly, preclinical studies found a direct connection between enteric ACh and propionate, linking this neurotransmitter to lipid homeostasis (192). Recently, a study found higher levels of intestinal Bacteroidetes in smokers, suggesting that nicotinic cholinergic signaling may have trophic effects on this phylum (162). Since smoking and body weight are known to be inversely related, and smoking cessation has been associated weight gain, targeting nicotinic receptors may comprise a new strategy for Bacteroidetes phylum restoration (193). In addition, as weight loss is a reported adverse effect of cholinesterase inhibitors, including donepezil and rivastigmine, these agents may help restore physiological levels of Bacteroidetes in chronic psychiatric patients (194).

## Conclusion

Psychotropic drugs-induced weight gain is marked by intestinal dysbiosis with Bacteroidetes phylum depletion. Propionate and indole, molecules that under normal circumstances are generated by Bacteroidetes, activate AhR and PXR xenobiotic receptors, modulating lipid and redox homeostasis. Bacteroidetes depletion by antibiotics, psychotropics or other drugs may deprive the host of these indispensable molecules, altering drug, lipid, and iron metabolism with subsequent weight gain. From this perspective, psychotropic drugs are indirect iron chelators as they lower the absorption of this biometal via gut microbes, decreasing body iron stores. The subsequent extracellular hypoferremia activates nutritional immunity with iron sequestration in enteric macrophages and IECs. Upregulated intracellular iron increases the risk of excessive ROS generation and the subsequent weight gain. Fortunately, obesity is a modifiable risk factor of general morbidity, therefore restoring the physiological levels of Bacteroidetes phylum by various strategies may attenuate or reverse the excess weight in chronic psychiatric patients. If validated, the biological markers described here, may offer the clinician an additional feed-back to estimate the imminence of weight-related complications.

## Author Contributions

All authors listed have made a substantial, direct and intellectual contribution to the work, and approved it for publication.

## Conflict of Interest

The authors declare that the research was conducted in the absence of any commercial or financial relationships that could be construed as a potential conflict of interest.

## Abbreviations

SSD: schizophrenia-spectrum disorders
OLZ: olanzapine
CZP: clozapine
RSP: risperidone
QTP: quetiapine
AhR: aryl hydrocarbon receptor
PXR: pregnane X receptor
FMT: fecal microbiota transplant
tMS: transcranial magnetic stimulation
IECs: intestinal epithelial cells
Trp: tryptophan
TLR9: toll like receptor 9
NTS: nucleus tractus solitaries
POMC: pro-opiomelanocortin
CART: cocaine-and amphetamine-regulated transcript
B. theta: Bacteroidetes thetaiotaomicron
VPA: valproic acid
DEPs: drug efflux pumps
cfDNA: cell free DNA
HIA: hydroxylamine-based indole assay
vBloc: vagal blocking device.
